# Clinically approved combination immunotherapy: Current status, limitations, and future perspective

**DOI:** 10.1016/j.crimmu.2022.05.003

**Published:** 2022-06-03

**Authors:** Ligong Lu, Meixiao Zhan, Xian-Yang Li, Hui Zhang, Danielle J. Dauphars, Jun Jiang, Hua Yin, Shi-You Li, Sheng Luo, Yong Li, You-Wen He

**Affiliations:** a Zhuhai Interventional Medical Center, Zhuhai Precision Medical Center, Zhuhai People's Hospital, Zhuhai Hospital Affiliated with Jinan University, Zhuhai, Guangdong Province, 519000, PR China; b First Affiliated Hospital, China Medical University, Shenyang, China; c Department of Immunology, Duke University Medical Center, Durham, NC, 27710, USA; d Tricision Biotherapeutic Inc, Jinwan District, Zhuhai, China; e Department of Biostatistics and Bioinformatics, Duke University Medical Center, Durham, NC, 27710, USA

**Keywords:** Combination immunotherapy, First-line therapy, Atezolizumab, Bevacizumab, HCC, Dose, Biomarker, Gene signature, Patient selection

## Abstract

Immune-checkpoint inhibitor-based combination immunotherapy has become a first-line treatment for several major types of cancer including hepatocellular carcinoma (HCC), renal cell carcinoma, lung cancer, cervical cancer, and gastric cancer. Combination immunotherapy counters several immunosuppressive elements in the tumor microenvironment and activates multiple steps of the cancer-immunity cycle. The anti-PD-L1 antibody, atezolizumab, plus the anti-vascular endothelial growth factor antibody, bevacizumab, represents a promising class of combination immunotherapy. This combination has produced unprecedented clinical efficacy in unresectable HCC and become a landmark in HCC therapy. Advanced HCC patients treated with atezolizumab plus bevacizumab demonstrated impressive improvements in multiple clinical endpoints including overall survival, progress-free survival, objective response rate, and patient-reported quality of life when compared to current first-line treatment with sorafenib. However, atezolizumab plus bevacizumab first-line therapy has limitations. First, cancer patients falling into the criteria for the combination therapy may need to be further selected to reap benefits while avoiding some potential pitfalls. Second, the treatment regimen of atezolizumab plus bevacizumab at a fixed dose may require adjustment for optimal normalization of the tumor microenvironment to obtain maximum efficacy and reduce adverse events. Third, utilization of predictive biomarkers is urgently needed to guide the entire treatment process. Here we review the current status of clinically approved combination immunotherapies and the underlying immune mechanisms. We further provide a perspective analysis of the limitations for combination immunotherapies and potential approaches to overcome the limitations.

## Highlights

• ICI-based combination immunotherapies become a 1st-line therapy for major cancers.
• Atezolizumab plus bevacizumab has produced unprecedented efficacy in HCC.
• Further optimization of patient selection, doses and biomarkers is needed.
• Potential approaches may overcome these limitations of combination immunotherapies.

## Graphical abstract

This review summarized the current status of clinically approved immune-checkpoint inhibitor-based combination immunotherapies. With several combination immunotherapies entering first-line treatment for major types of cancer including hepatocellular carcinoma (HCC), renal cell carcinoma, lung cancer, cervical cancer, and gastric cancer, it is critical to understand the limitations of these clinically approved immunotherapies. Using the combination of anti-PD-L1 antibody, atezolizumab, plus the anti-vascular endothelial growth factor antibody, bevacizumab, as an example, we discussed the limitations of this first-line treatment for advanced HCC. First, cancer patients falling into the criteria for the combination therapy may need to be further selected to reap benefits while avoiding some potential pitfalls. Second, the treatment regimen of atezolizumab plus bevacizumab at a fixed dose may require adjustment for optimal normalization of the tumor microenvironment to obtain maximum efficacy and reduce adverse events. Third, utilization of predictive biomarkers is urgently needed to guide the entire treatment process. We further provide potential approaches to overcome the limitations.As shown below on the limitations and potential approaches of first-line atezolizumab plus bevacizumab combination therapy for HCC, subgroups of advanced HCC patients falling into the treatment criteria of first-line atezolizumab plus bevacizumab therapy may be further selected based on superior or nonsuperior OS when compared to first-line sorafenib therapy. The dose of atezolizumab and bevacizumab may be adjusted according to clinical studies in the indicated ranges. A composite biomarker consists of three types is likely needed to guide patient selection and therapeutic course.

**Figure:**
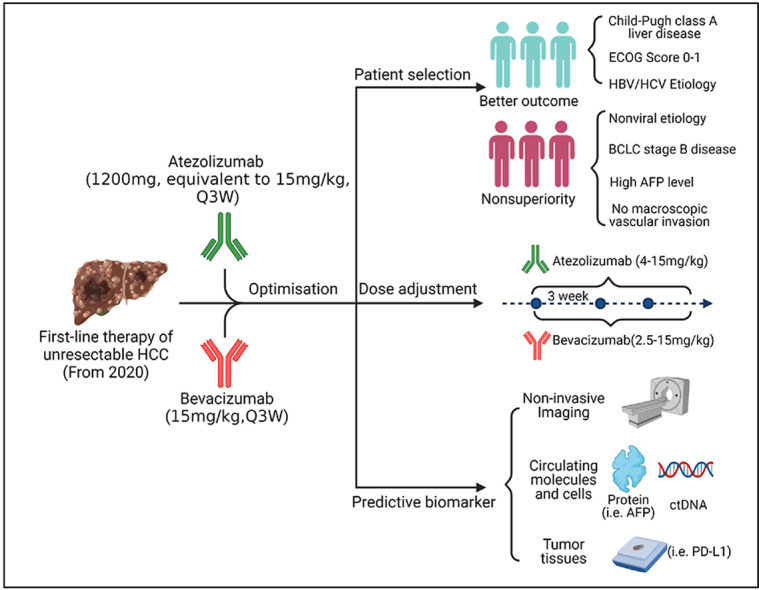

## List of abbreviations

AFP: alpha-fetoprotein
ECOG: Eastern Cooperative Oncology Group;
HCC: hepatocellular carcinoma
HR: hazard ratio
ICI: immune checkpoint inhibitor
mAb: monoclonal antibody
mRCC: metastatic renal cell carcinoma
NAFLD: non-alcoholic fatty liver disease
NASH: non-alcoholic steatohepatitis
NSCLC: non-squamous non-small cell lung cancer
ORR: objective response rate
OS: overall survival
PIVKA-II: protein induced by vitamin K absence-II
PFS: progression free survival
Q2/3/4W: once every two/three/four week
TME: tumor microenvironment
VEGF: vascular endothelial growth factor

## 1 Introduction

Immune-checkpoint inhibitor (ICI)-based immunotherapy has revolutionized cancer treatment and has become the standard of care for many cancer patients (Sharma et al., 2021). Although single agent ICI treatment produces durable responses in advanced stage cancer patients, only a small fraction of patients benefits and relapse/recurrence frequently occurs due to various resistance mechanisms (Sharma et al., 2021). The limitation of ICI-monotherapy in most cancer patients is due to the complex tumor microenvironment (TME) with multiple layers of immunosuppressive factors in each step of the cancer-immunity cycle (Chen and Mellman, 2013). To achieve effective tumor control and eradication, three levels of immunotherapeutic competence are required. First, all three cellular components of adaptive immunity, CD4^+^ T, CD8^+^ T, and B lymphocytes, are activated to become antigen-specific effector cells with full access to tumor cells. Second, the immunosuppressive factors in the tumor microenvironment, represented by PD-L1 and others, are neutralized. Third, the tumor cells express relevant targets recognized by the effectors and are sensitive to immune cell mediated killing (Lu et al., 2021a). Thus, combination immunotherapies targeting multiple steps of the cancer-immunity cycle are necessary to achieve long-term efficacy in a large fraction of cancer patients (Yap et al., 2021; Meric-Bernstam et al., 2021). As of May 10, 2022, the US Food and Drug Administration has approved 35 ICI-based combination immunotherapies (Table 1). The clinically approved combinations consist of ICIs (anti-PD-1 plus anti-CTLA4), ICI plus chemotherapy, and ICI plus targeted therapy in hepatocellular carcinoma (HCC), non-small cell lung cancer (NSCLC), renal cell carcinoma (RCC), melanoma, breast cancer, urothelial carcinoma, gastric cancer, cervical cancer, and endometrial carcinoma (Table 1).

**Table 1:** List of 35 FDA-approved combination immunotherapies as of May 10, 2022.

FDA approval date	Tumor type	Combination agent
10/01/2015	BRAF^WT^ metastatic melanoma	Nivolumab + Ipilimumab
01/23/2016	Metastatic melanoma across BRAF status	Nivolumab + Ipilimumab
05/10/2017	First-line metastatic NSCLC	Pembrolizumab + Pemetrexed + Carboplatin
02/16/2018	Stage III NSCLC	Durvalumab + Chemoradiation
04/16/2018	First-line intermediate or poor-risk advanced RCC	Nivolumab + Ipilimumab
07/10/2018	MSI-H or dMMR metastatic CRC	Nivolumab + Ipilimumab
08/20/2018	Metastatic nonsquamous NSCLC	Pembrolizumab + Pemetrexed + Chemotherapy
10/30/2018	First-line metastatic squamous NSCLC	Pembrolizumab + Chemotherapy
12/06/2018	First-line NSCLC	Atezolizumab + bevacizumab, paclitaxel and carboplatin
03/08/2019	Metastatic TNBC	Atezolizumab + Nabpaclitaxel
03/18/2019	Extensive-stage SCLC	Atezolizumab + Carboplatin + Etoposide
04/19/2019	First-line advanced RCC	Pembrolizumab + Axitinib
05/14/2019	First-line advanced RCC	Avelumab + Axitinib
06/11/2019	HNSCC	Pembrolizumab + Chemotherapy
06/17/2019	Metastatic SCLC	Pembrolizumab + Chemotherapy
09/17/2019	Advanced endometrial carcinoma	Pembrolizumab + Lenvatinib
12/03/2019	First-line Metastatic NSCLC without EGFR/ALK aberrations	Atezolizumab + Nab-paclitaxel + Carboplatin
03/10/2020	Advanced HCC after sorafenib	Nivolumab + Ipilimumab
03/27/2020	Extensive-stage SCLC	Durvalumab + Etoposide + either Carboplatin or Cisplatin
05/15/2020	First-line mNSCLC (PD-L1 tumor expression ≥1%)	Nivolumab + Ipilimumab
05/26/2020	First-line Metastatic NSCLC	Nivolumab + Ipilimumab +2 cycles of platinum-doublet chemotherapy
05/29/2020	First-line unresectable HCC	Atezolizumab + Bevacizumab
06/30/2020	Locally advanced or metastatic urothelial carcinoma	Avelumab + Chemotherapy
07/30/2020	BRAF V600 unresectable or metastatic melanoma	Atezolizumab + Cobimetinib + Vemurafenib
10/02/2020	First-line malignant pleural mesothelioma	Nivolumab + Ipilimumab
11/13/2020	Locally recurrent unresectable or metastatic TNBC	Pembrolizumab + Chemotherapy
01/22/2021	First-line advanced RCC	Nivolumab + Cabozantinib
03/22/2021	Esophageal or GEJ carcinoma	Pembrolizumab + Chemotherapy
04/16/2021	Metastatic gastric cancer and esophageal adenocarcinoma	Nivolumab + fluoropyrimidine- and platinum-containing chemotherapy
05/05/2021	First-line HER2-positive gastric cancer	Pembrolizumab + Trastuzumab + Chemotherapy
07/21/2021	Advanced endometrial carcinoma	Pembrolizumab + Lenvatinib
07/26/2021	High-risk early-stage TNBC	Pembrolizumab + Chemotherapy
08/10/2021	First-line advanced RCC	Pembrolizumab + Lenvatinib
10/13/2021 03/04/2022	First-line cervical cancer Neoadjuvant resectable NSCLC	Pembrolizumab + Chemotherapy ± Bevacizumab Nivolumab + Chemotherapy

**Note:** Dates are listed as month/day/year. GEJ: gastroesophageal junction; HNSCC: head and neck squamous cell carcinoma; SCLC: small cell lung cancer; TNBC: triple negative breast cancer. Source: https://www.fda.gov/drugs/resources-information-approved-drugs/oncology-cancer-hematologic-malignancies-approval-notifications.

Dates are listed as month/day/year. CRC: colorectal cancer; GEJ: gastroesophageal junction; HNSCC: head and neck squamous cell carcinoma; NSCLC: non-small cell lung cancer. SCLC: small cell lung cancer; TNBC: triple negative breast cancer.

Among the approved combination immunotherapies, ICI plus antiangiogenic agents warrants special attention as several combinations have become first-line therapy for major types of cancer HCC, RCC, NSCLC, and cervical cancer (Table 1). Three first-line ICIs plus antiangiogenic agents (pembrolizumab + axitinib, avelumab + axitinib, and nivolumab + cabozantinib) have dramatically changed the landscape of RCC treatment by prolonging patient survival over targeted therapy (Rini et al., 2019a; Motzer et al., 2019; Choueiri et al., 2021). Moreover, atezolizumab plus bevacizumab with chemotherapeutic agent carboplatin and paclitaxel were approved as first-line treatment for NSCLC (Socinski et al., 2018). Recently, the phase III IMbrave150 trial combining atezolizumab plus bevacizumab as first-line treatment for unresectable HCC has shown remarkable clinical efficacy (Finn et al., 2020a). This unprecedented clinical efficacy in liver cancer treatment has generated tremendous excitement in the field and is heralded as a new era, a landmark, and a novel breakthrough (Kelley, 2020; Kudo, 2020; Sangro et al., 2021; Castet et al., 2021). With the approval of atezolizumab plus bevacizumab as first-line treatment for advanced HCC, this combination therapy has been rapidly adopted as the preferred first-line treatment for most patients with advanced HCC by American Society of Clinical Oncology guidelines (Gordan et al., 2020) and National Comprehensive Cancer Network guidelines (Benson et al., 2021).

Like all treatments, combination immunotherapies have limitations and face important challenges. For example, it is unknown how different RCC patients will respond to the different first-line treatments of ICIs plus antiangiogenic agents and whether these combinations are the best option for all RCC patients (Rossi et al., 2021). Similarly, personalized treatment approaches with robust predictive biomarkers are urgently needed to identify NSCLC patients responsive to the atezolizumab/bevacizumab/carboplatin/paclitaxel combination immunotherapy (Wang et al., 2021). Given the remarkable efficacy and the preferred status of atezolizumab plus bevacizumab as first-line choice for advanced HCC patients, we use this combination immunotherapy as an example to discuss the current status, potential pitfalls and future perspective on combination immunotherapies. There are three major limitations of atezolizumab plus bevacizumab as first-line therapy for advanced HCC patients. First, advanced HCC patients falling into the criteria for the combination therapy may need to be further selected to reap benefits while avoiding some potential pitfalls. Second, the treatment regimen of atezolizumab plus bevacizumab at a fixed dose may require adjustment for optimal normalization of the TME in HCC to obtain maximum efficacy and reduce adverse events. Third, utilization of predictive immune/vascular biomarkers is urgently needed to guide the entire treatment process. Here we provide a detailed discussion on these issues and potential approaches to overcome the limitations.

## 2 Features of atezolizumab plus bevacizumab combination immunotherapy and underlying immune mechanisms

HCC is a lethal cancer with an 18% 5-year survival rate for patients at all stages (Jemal et al., 2017; Villanueva, 2019). Systemic treatments including single-agent multikinase inhibitors and ICIs for advanced-stage HCC have shown only suboptimal clinical efficacies (Finn and Zhu, 2021). Single-agent therapy of anti-PD-1 mAb nivolumab in CheckMate 040 or pembrolizumab in KEYNOTE-224 studies demonstrated a durable 15–20% objective response rate (ORR) in sorafenib-naïve or -experienced HCC patients (El-Khoueiry et al., 2017; Zhu et al., 2018). The anti-PD-L1 mAb atezolizumab as a first-line single-agent also achieved a 17% ORR in patients with advanced HCC in the phase Ib GO30140 study (Lee et al., 2020). In the subsequent phase III CheckMate-459 study, nivolumab as a first-line therapy produced a median overall survival (mOS) of 16.4 vs. 14.8 months (hazard ratio, HR 0.85; 95% CI, 0.72–1.00; p = 0.0522) (Yau et al., 2019; Sangro et al., 2020a). Furthermore, mOS in patients with PD-L1 > 1% was longer in nivolumab treated HCC patients when compared to those treated with sorafenib (16.1 months [95% CI, 8.4e22.3] vs 8.6 months [95% CI, 5.7e 16.3], respectively). Importantly, patients with virus-associated HCC also had longer mOS in nivolumab treated group vs sorafenib group (17.5 vs 12.7 months; HR, 0.72 [95% CI, 0.51e1.02] for HCV and 16.1 vs 10.4 months; HR 0.79 [95% CI, 0.59e1.07] for HBV, respectively) (Sangro et al., 2020a). The phase III KEYNOTE-240 study testing pembrolizumab as a second-line therapy in patients with advanced HCC yielded an mOS of 13.9 months vs. 10.6 months for placebo (HR, 0.781; 95% CI, 0.611–0.998; p = 0.0238) (Finn et al., 2020b). Median PFS for pembrolizumab was 3.0 months vs 2.8 months at final analysis (HR, 0.718; 95% CI, 0.570–0.904; p = 0.0022) (Finn et al., 2020b). Although both phase III studies did not reach pre-specified statistical criteria, it is clear that single-agent nivolumab as a first-line and pembrolizumab as a second-line therapy conferred clinically meaningful survival benefits. Thus, ICI-based monotherapy of advanced HCC has the potential to be further improved by combining agents.

**Fig. 1:**
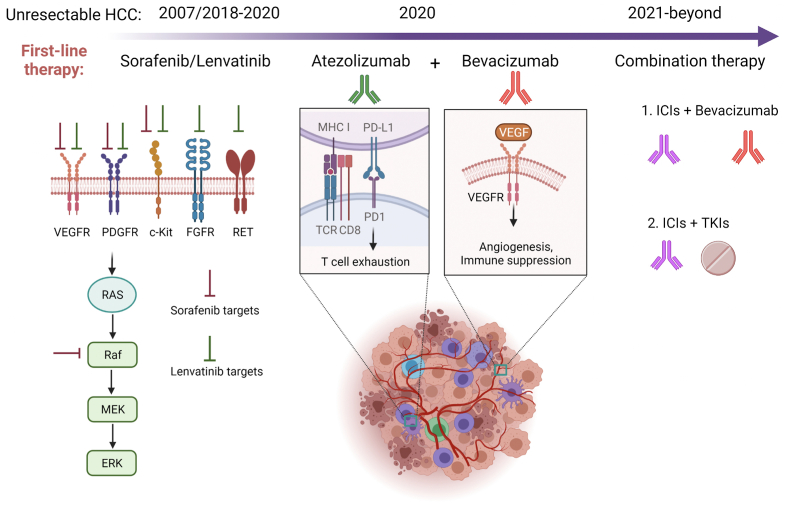
**Mechanism of current and future first-line therapy for HCC.** Sorafenib and lenvatinib are multikinase inhibitors primarily targeting tumor cells and endothelial cells. Atezolizumab blocks PD-1 engagement of PD-L1 while bevacizumab blocks VEGF interaction with its receptor in immune cells as well as endothelial cells. Clinical trials with different combinations of ICIs (anti-PD-1/L1 and anti-CTLA-4) plus bevacizumab or bevacizumab biosimilar (IBI305) and ICIs plus TKIs (lenvatinib, cabozantinib, apatinib) are under active investigation.

There is a strong rationale to combine ICIs with antiangiogenic agents in combination immunotherapy (Fukumura et al., 2018; Morse et al., 2019; Hack et al., 2020; Pinter et al., 2021; Chambers et al., 2021)(Fig. 1). Vascular endothelial growth factor (VEGF) not only promotes angiogenesis in tumors but also fundamentally programs an immunosuppressive TME by recruiting and inducing immunosuppressive cells including regulatory T cells, tumor-associated macrophages, and myeloid-derived suppressor cells (Kudo, 2020; Morse et al., 2019; Zhu et al., 2011). Furthermore, VEGF inhibits dendritic cell differentiation and maturation as well as effector T cell proliferation, thereby impairing T cell priming and killing of tumor targets (Gabrilovich et al., 1998; Oyama et al., 1998; Mimura et al., 2007). Thus, blocking VEGF signaling may not only inhibit intratumoral angiogenesis to normalize the tumor vasculature but also re-program the immunosuppressive TME into an immune-stimulating one (Fig. 1). Indeed, blocking VEGF with bevacizumab in triple-negative breast cancer patients improved tumor infiltration of mature DCs and effector T cells (Boucher et al., 2021). In mouse tumor models, anti-VEGF treatment resulted in more mature DCs and less exhausted T cells intratumorally as well as reprograming of immunosuppressive M2-like macrophages into immune stimulatory M1-like macrophages (Malo et al., 2018; Huang et al., 2012). An early study demonstrated administration of bevacizumab in an orthotopic HCC mouse model decreased tumor microvessel density and prolonged time to progression in tumor-bearing mice (Finn et al., 2009). Two phase II studies testing single agent bevacizumab therapy in unresectable HCC patients showed 13–14% ORRs, suggesting that VEGF is a valid target for HCC treatment (Siegel et al., 2008; Boige et al., 2012).

Several important features from the IMbrave150 study have made atezolizumab plus bevacizumab the premier choice of first-line therapy for patients with advanced HCC. First, atezolizumab plus bevacizumab combination therapy readily showed superiority to sorafenib in OS. In updated OS data reports, the mOS with atezolizumab plus bevacizumab vs. sorafenib was 19.2 and 13.4 months respectively (HR, 0.66; 95% CI, 0.52–0.85; p = 0.0009) (Finn et al., 2021; Cheng et al., 2022). Furthermore, median PFS in patients treated with atezolizumab plus bevacizumab was 6.9 months vs. 4.3 months for sorafenib (HR, 0.59; 95% CI, 0.47–0.76; p < 0.001) (Cheng et al., 2022). There is no direct comparison of clinical efficacy between atezolizumab plus bevacizumab and lenvatinib, another first line treatment for advanced HCC (Kudo et al., 2018). However, indirect comparison showed that atezolizumab plus bevacizumab has a superior OS over lenvatinib (HR, 0.59–0.63) as first-line therapy for advanced HCC (Sonbol et al., 2020; Casadei-Gardini et al., 2021; Vogel et al., 2021). Thus, atezolizumab plus bevacizumab provides an OS advantage over both sorafenib and lenvatinib in systemic treatment of naïve patients with advanced HCC and is expected to be accepted by physicians as the preferred first-line choice.

Second, atezolizumab plus bevacizumab combination therapy produced clinically meaningful benefits in patient-reported quality of life, functioning, and disease symptoms (Finn et al., 2020a; Galle et al., 2021). Median time to deterioration for quality of life was 11.2 months with atezolizumab plus bevacizumab vs. 3.6 months with sorafenib (HR, 0.63; 95% CI, 0.46–0.85) (Finn et al., 2020a). Remarkably, atezolizumab plus bevacizumab combination therapy reduced the risk of deterioration on all prespecified generic cancer symptoms including appetite loss, diarrhea, fatigue, and pain (Galle et al., 2021). Thus, in comparison to sorafenib, the combination therapy offers an overall better quality of life during disease treatment and is therefore likely preferred by patients as well.

Third, atezolizumab plus bevacizumab combination therapy yielded a deeper and broader disease response when compared to sorafenib (Finn et al., 2020a, 2021; Cheng et al., 2022; Salem et al., 2021; Qin et al., 2021). At the depth front, atezolizumab plus bevacizumab generated better ORR (with both improved complete response and partial response), duration of response, and depth of response than sorafenib when evaluated by either RECIST1.1 or HCC-modified RECIST (mRECIST). It is worth noting that a striking 25 of 326 patients (7.7% per RECIST1.1) or 39 of 325 patients (12.0% per mRECIST) have achieved complete response (Finn et al., 2021). Equally impressively, atezolizumab plus bevacizumab combination therapy provided better survival than sorafenib in most of the subgroup analyses including age, sex, geographic region, Eastern Cooperative Oncology Group Performance Status (ECOG) score, AFP <400 ng/ml, presence of macrovascular invasion and extrahepatic spread, HBV and HCV etiologies, and prior local therapies (Finn et al., 2020a; Cheng et al., 2022; Qin et al., 2021; Li et al., 2020). Furthermore, the combination therapy also provided better ORR for HCC patients with large tumor sizes (≥3 cm) (Salem et al., 2021). Together with a manageable safety profile, atezolizumab plus bevacizumab combination immunotherapy is an indisputable first-line choice for patients with advanced HCC. The IMmotion151 phase III study also supports atezolizumab plus bevacizumab combination immunotherapy as a first-line treatment option for selected patients with advanced RCC (Rini et al., 2019b). Similar combinations using ICIs plus anti-VEGF/R signaling inhibitors have either been tested in phase I/II trials or under active phase III trials in HCC (Sangro et al., 2021; Finn et al., 2020c; Xu et al., 2021; Mei et al., 2021; Yau et al., 2020; Ren et al., 2021). Thus, it is highly anticipated that additional effective combination therapies will come of these trials for advanced HCC (Fig. 1).

## 3 Patient selection for atezolizumab plus bevacizumab first-line therapy

**Fig. 2:**
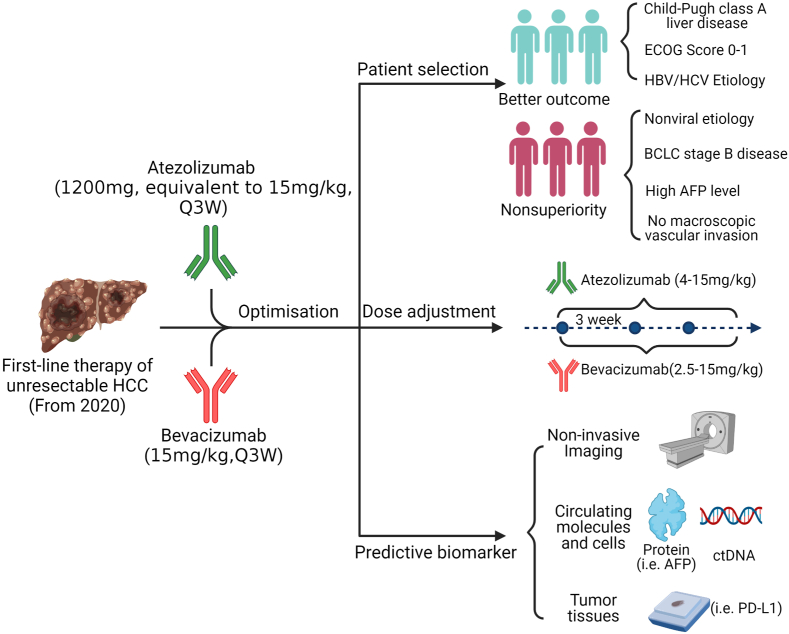
**Limitations and potential approaches of first-line atezolizumab plus bevacizumab combination therapy for HCC.** Subgroups of advanced HCC patients falling into the treatment criteria of first-line atezolizumab plus bevacizumab therapy may be further selected based on superior or nonsuperior OS when compared to first-line sorafenib therapy. The dose of atezolizumab and bevacizumab may be adjusted according to clinical studies in the indicated ranges. A composite biomarker consisting of three types is likely needed to guide patient selection and therapeutic course.

A major challenge for combination immunotherapy is whether the cancer patient population who fit the inclusion criteria shall all receive the approved therapy. Patients with advanced HCC recommended by current guidelines for atezolizumab plus bevacizumab first-line therapy are a well-selected group (Gordan et al., 2020; Benson et al., 2021). The recommendation is based on IMbrave150 study inclusion criteria including Child-Pugh class A liver disease, ECOG score 0–1, following management of esophageal varices, no contraindications to atezolizumab and/or bevacizumab, no myocardial infarction or stroke within 3 months, and no coinfection of HBV and HCV (Finn et al., 2020a; Gordan et al., 2020; Benson et al., 2021). Contraindications to anti-angiogenetic therapy alone may exclude 10–15% of HCC patients (Sangro et al., 2021; Pelizzaro et al., 2019). Thus, an important question of whether even this well-selected advanced HCC patient population should all received atezolizumab plus bevacizumab as first-line therapy arises (Fig. 2). In subgroup analysis of survival benefit, although it is clear that atezolizumab plus bevacizumab offers a survival advantage over sorafenib to most subgroups, as discussed above, several subgroups of patients, including BCLC stage B disease, nonviral etiology, high AFP levels, absence of macroscopic vascular invasion and extrahepatic spread, no prior local therapy, and PD-L1 score (TC and IC) < 1% appear to have similar OS and PFS in both groups (Finn et al., 2020a; Cheng et al., 2022)(Fig. 2).

Several issues should be considered in deciding whether atezolizumab plus bevacizumab or sorafenib/lenvatinib is chosen as first-line treatment for these subgroups of HCC patients. First, does atezolizumab plus bevacizumab combination therapy yield better quality of life than sorafenib/lenvatinib treatment in these subgroups? Second, does atezolizumab plus bevacizumab combination therapy as first-line treatment provide a better foundation/disease condition for eventual second-line therapy than sorafenib/lenvatinib in these subgroups? Third, but not least important, is the affordability issue. Recent cost-effectiveness analyses indicate that compared to sorafenib, atezolizumab plus bevacizumab first-line therapy has an incremental cost-utility ratio of $145,546.21 per quality-adjusted life year in China and $168,030.21-$179,729 per quality-adjusted life year in the United States, well above the willing-to-pay threshold (Su et al., 2021; Wen et al., 2021; Chiang et al., 2021). Thus, atezolizumab plus bevacizumab combination therapy at its current price is not a cost-effective option when compared to sorafenib. For the above subgroup patients with similar OS between atezolizumab plus bevacizumab and sorafenib, cost-effective sorafenib may still be chosen as first-line treatment.

Special attention to the etiology of advanced HCC may be needed in deciding which first-line treatment to use. Patients with HCC of nonviral etiology treated with the combination therapy had an HR of 0.80 (95% CI, 0.55–1.17) on OS when compared to sorafenib (Cheng et al., 2022). This group accounted for 30.5% (153/501) of all treated patients. Recent meta-analyses of randomized controlled trials of HCC demonstrated that ICI-based immunotherapy is less effective in nonviral HCC (HR, 0.92; 95% CI, 0.79–1.06) than HBV- or HCV-related HCC (HR, 0.64; 95% CI, 0.5–0.83 or 0.48–0.94) when compared with the OS in standard-care groups (Haber et al., 2021; Pfister et al., 2021). In contrast, tyrosine kinase inhibitor/anti-VEGF therapies have similar treatment efficacy with HRs for OS at 0.81 (95% CI, 0.71–0.92) for HCC of viral etiology and 0.82 (95% CI,0.67–1.01) for that of nonviral etiology (p = 0.8828) (Haber et al., 2021). Unexpectedly, patients with non-alcoholic steatohepatitis (NASH)-driven HCC receiving ICI-based immunotherapy had much reduced OS (Pfister et al., 2021). In two small cohorts of advanced HCC patients treated with anti-PD-1/L1, the patients with non-alcoholic fatty liver disease (NAFLD) etiology had an mOS of 5.4–8.8 months while the patients with other etiologies had an mOS of 11.0–17.7 months (p = 0.023–0.034) (Pfister et al., 2021). The seemingly detrimental effect on OS of patients with NASH- or NAFLD-HCC by anti-PD-1/L1 antibody treatment may be related to impaired immune surveillance by exhausted hepatic CD8^+^PD-1^+^ T cells (Pfister et al., 2021). In the IMbrave150 study, the 153 nonviral HCC patients were further divided into alcohol-related, other, and unknown without specifying NASH or NAFLD cause. Given the current data, it may be prudent to use sorafenib/lenvatinib for HCC of nonviral etiology, especially for NASH/NAFLD etiology, as first-line treatment. Thus, one-third of advanced HCC patients fitting the criteria for atezolizumab plus bevacizumab first-line therapy may be better suited for sorafenib/lenvatinib treatment (Fig. 2). As NAFLD and NASH are rapidly increasing globally and becoming a leading cause of HCC in Western countries due to obesity-associated metabolic syndrome (Younossi et al., 2018; Llovet et al., 2021), the efficacy (or lack thereof) of atezolizumab plus bevacizumab combination therapy in this subgroup of HCC patients urgently needs to be firmly established.

Why does atezolizumab plus bevacizumab first-line therapy exhibit better clinical activity in HCC of viral etiology than that of non-viral etiology? An initial hypothesis was put forward that viral antigens expressed by tumor cells may serve as potent immunogens to stimulate antigen-specific T lymphocytes and enhance antitumor immunity (Ho et al., 2020). However, analyses of the immune landscapes in the TME of viral- and non-viral HCC revealed surprisingly high similarities (Ho et al., 2020; Foerster et al., 2018; Lim et al., 2019). Several potential mechanisms might account for the superior clinical activity by atezolizumab plus bevacizumab first-line therapy in HCC with viral etiology. First, small but critical immune cell populations may be activated by the combination therapy in viral HCC. For example, CD8^+^ resident memory T cells and Tregs were enriched in HBV-related HCC while Tim3^+^CD8^+^ T cells and CD244^+^ NK cells were enriched in non-viral related HCC (Lim et al., 2019). Second, the TME of viral and non-viral HCC may differ in their response to bevacizumab when it is used in combination. Thus, comprehensive studies on all components of the liver tissues from viral vs non-viral HCC undergoing atezolizumab plus bevacizumab therapy are needed to elucidate the underlying cellular and molecular mechanisms and provide better biomarkers for patient selection.

## 4 Treatment regimen for atezolizumab plus bevacizumab first-line therapy to normalize the tumor vasculature

Clinical efficacy critically relies upon the specific treatment regimen of ICIs plus antiangiogenic agents. However, the optimal doses for combination immunotherapies are largely unknown. The recommended treatment regimen for atezolizumab plus bevacizumab first-line therapy in patients with advanced HCC is atezolizumab 1,200 mg plus bevacizumab 15 mg/kg once every three weeks (Q3W) by intravenous infusion (Finn et al., 2020a; Gordan et al., 2020; Benson et al., 2021). This fixed dose for atezolizumab and bevacizumab is based on the IMbrave150 study, which did not test dose variation. The same regimen was also used in the IMmotion151 phase III trial treating metastatic RCC (Rini et al., 2019b). As discussed extensively by others (Fukumura et al., 2018; Morse et al., 2019; Hack et al., 2020; Pinter et al., 2021), bevacizumab likely blocks multiple tumorigenic functions of VEGF, reversing angiogenesis and immunosuppression within the TME of HCC. One of the major antitumor functions exerted by anti-angiogenic therapy is to induce tumor vasculature normalization (Jain, 2001, 2014; Martin et al., 2019) (Fig. 1). Normalized vasculature alleviates hypoxia, increases tumor perfusion, and restores immune cell infiltration (Jain, 2014). However, the benefit of anti-angiogenic-induced tumor vascular normalization critically depends on the dose and time of treatment (Pinter et al., 2021; Jain, 2014). High-dose anti-VEGF causes excess vessel pruning and decreased perfusion with increased hypoxia. To complicate things further, ICI-based therapy itself also has tumor vasculature normalization function (Tian et al., 2017; Zheng et al., 2018). It was shown in mouse tumor models that anti-CTLA4 and anti-PD-1 can induce tumor vessel normalization through activation of effector CD4^+^ or CD8^+^ T lymphocytes (Tian et al., 2017; Zheng et al., 2018). Atezolizumab is likely to have similar vasculature normalization function by blocking PD1-PD-L1 interaction and inducing activation of effector CD8^+^ T lymphocytes (Herbst et al., 2014; Powles et al., 2014). Therefore, an important unanswered question regarding the regimen of atezolizumab plus bevacizumab is whether the doses of these two drugs that both have vasculature normalizing functions are optimal, without causing excess vessel pruning in HCC tumors.

Bevacizumab is prescribed at several doses clinically in combination with chemotherapy and immunotherapy (Genentech, 2021). Except in metastatic colorectal cancer (mCRC) at three different regimens of 5 mg/kg or 10 mg/kg Q2W (2.5 or 5 mg/kg/week dose equivalent), and 7.5 mg/kg Q3W (2.5 mg/kg/week dose equivalent) in combination with different chemotherapies, all other cancer types including HCC, non-squamous non-small cell lung cancer (NSCLC), metastatic renal cell carcinoma (mRCC), cervical cancer, ovarian/fallopian tube, and primary peritoneal cancers use 15 mg/kg Q3W (5 mg/kg/week dose equivalent) in combination with chemotherapeutic and immunotherapeutic agents (Genentech, 2021). Numerous clinical studies have reported the antitumor efficacy of high-vs. low-dose bevacizumab alone or in combination therapy against several major cancer types (Falk et al., 2015). Most studies lacked sufficient power to draw definitive conclusions. Nevertheless, it appears that high-dose bevacizumab (5 mg/kg/week dose equivalent) yields clinical benefit in renal and lung cancers while low-dose bevacizumab (≤3 mg/kg/week dose equivalent) yields clinical benefit in mCRC and glioblastoma (Falk et al., 2015). Thus, different cancer types may require different doses of bevacizumab to maximize clinical efficacy.

Prior to the IMbrave150 trial, bevacizumab was extensively investigated in clinical studies of HCC therapy as a single-agent or combined with chemotherapeutic agents (Siegel et al., 2008; Boige et al., 2012; Zhu et al., 2006; Thomas et al., 2009, 2018; Hsu et al., 2010, 2013; Sun et al., 2011; Philip et al., 2012; Kaseb et al., 2012; Yau et al., 2012; Buijs et al., 2013; Govindarajan et al., 2013; Knox et al., 2015; Hubbard et al., 2017). Ten of the 15 phase II trials, above, used bevacizumab at 10 mg/kg Q2W (5 mg/kg/week dose equivalent) (Siegel et al., 2008; Boige et al., 2012; Zhu et al., 2006; Thomas et al., 2009, 2018; Philip et al., 2012; Kaseb et al., 2012; Yau et al., 2012; Buijs et al., 2013; Knox et al., 2015). The other 5 phase II trials used different treatment regimens ranged from 1.25 to 5 mg/kg/week dose equivalent including 2.5 mg/kg Q2W (Hubbard et al., 2017), 5 mg/kg Q3W (Sun et al., 2011), 5 mg/kg Q2W (Siegel et al., 2008; Boige et al., 2012; Hsu et al., 2013), 7.5 mg/kg Q3W (Hsu et al., 2010), and 15 mg/kg Q3W (Govindarajan et al., 2013). Thus, IMbrave150 used a high-dose bevacizumab at 5 mg/kg/week dose equivalent that is not only tested and approved by the FDA in other cancer types but also extensively tested in HCC as a single-agent or in combination. Furthermore, the recommended 15 mg/kg Q3W infusion of bevacizumab is conveniently timed together with atezolizumab infusion at 1,200 mg Q3W. Two phase II trials tested the clinical efficacy of low-dose (5 mg/kg Q2W) vs. high-dose (10 mg/kg Q2W) bevacizumab as a single-agent in patients with advanced HCC (Siegel et al., 2008; Boige et al., 2012). The Siegel study showed that patients receiving low-dose (n = 12) and high-dose (n = 34) bevacizumab had mOS at 15.1 and 12.2 months, respectively (p = 0.64) (Siegel et al., 2008). The Boige study reported a 16-week disease control rate of 39% for the low-dose group (n = 25) and 45% for the high-dose group (n = 23) (Boige et al., 2012). Although both studies had relatively small sample sizes and were not sufficiently powered, these data suggest that low-dose bevacizumab at < 5 mg/kg/week dose equivalent may have similar antitumor activity to high-dose bevacizumab at 5 mg/kg/week dose equivalent in HCC. Thus, it is worth testing a low dose of bevacizumab in the first-line combination treatment, not only to reduce adverse events, but also to avoid potentially excessive vessel pruning when combined with atezolizumab (Fig. 1, Fig. 2).

Atezolizumab is prescribed in three regimens at 840 mg Q2W, 1200 mg Q3W, and 1680 mg Q4W (400–420 mg/week dose equivalent) for all FDA approved indications, including HCC, urothelial carcinoma, NSCLC, metastatic triple-negative breast cancer, small cell lung cancer, and melanoma (Genentech: Tecentriq, 2021). A preclinical pharmacokinetics and pharmacodynamics study demonstrated that the exposure area under the serum drug concentration-time curve from time 0 to day 7 (AUC_0-7_) of atezolizumab (MPDL3280A) is proportional between 5 and 20 mg/kg, and ∼96% saturation of PD-L1 can be achieved at ∼0.5 μg/ml in monkeys (Deng et al., 2016). In a large-scale phase I trial, it was found that atezolizumab had antitumor activity across a dose range of 1–20 mg/kg Q3W (Herbst et al., 2014). The minimum dose to maintain a trough serum concentration at steady state (C_trough, ss_) ≥ 6 μg/ml in ≥90% of patients is estimated at 4 mg/kg Q3W (Herbst et al., 2014; Deng et al., 2016). It was decided that 15 mg/kg Q3W and the equivalent fixed dose of 1,200 mg Q3W were sufficient to maintain a target trough concentration of 6 μg/ml and was the chosen dose in clinical development as a monotherapy (Herbst et al., 2014; Powles et al., 2014). Therefore, the optimal dose of atezolizumab when used in combination with bevacizumab is unknown and needs to be investigated in prospective trials. Given the large margin of prescribed atezolizumab (15 mg/kg Q3W vs. 4 mg/kg Q3W required for C_trough, ss_ ≥ 6 μg/ml), it is tempting to speculate that the dose of atezolizumab may be reduced when in combination with bevacizumab.

## 5 Predictive immune/vascular biomarkers for atezolizumab plus bevacizumab first-line therapy

Combination immunotherapies using ICIs plus antiangiogenic agents may have unique biomarkers for prediction and patient selection. A sizable portion of patients with advanced HCC receiving atezolizumab plus bevacizumab first-line therapy did not benefit from this treatment (Finn et al., 2020a). With the high cost of the combination regimen, it is essential to have predictive biomarker(s) to guide treatment decisions prior to and during therapy. The early separation of OS and PFS curves (within 2 months) between the two groups of patients treated with the combination therapy or sorafenib in the IMbrave150 trial is a very encouraging sign, suggesting the potential to identify predictive biomarker(s) either before the initiation of therapy or during the early phase of treatment (Finn et al., 2020a; Qin et al., 2021). Indeed, biomarker analysis of tumor samples taken prior to treatment in the phase Ib GO30140 trial revealed that pre-existing immunity in baseline tumor gene expression is associated with clinical response and longer PFS in advanced HCC patients treated with atezolizumab plus bevacizumab first-line therapy (Zhu et al., 2020). Specifically, high expression of PD-L1 and T lymphocyte effector signature (granzyme B, perforin 1, and CXCL9) was associated with better outcomes (HR = 0.42; 95% CI, 0.25–0.72; p < 2.1 × 10^−5^ for PD-L1) (HR = 0.46; 95% CI, 0.27–0.78; p < 0.0004 for T cell signature). Furthermore, high expression of Treg, myeloid inflammation, and MDSC gene signatures, and VEGF receptor 2 in tumor tissues was associated with longer PFS in patients treated with atezolizumab plus bevacizumab than those treated with atezolizumab monotherapy (Zhu et al., 2020). If validated in prospective trials, these biomarkers will be important for patient selection prior to treatment initiation. However, aside from the lack of validation, procurement of tumor tissue samples poses a practical challenge. Furthermore, biomarkers informative of early responses are needed to guide timely regimen change to second-line therapies. Three types of biomarkers detected by differentially invasive procedures can be used to guide atezolizumab plus bevacizumab first-line therapy in patients with advanced HCC (Fig. 2).

The first type of biomarker is found by non-invasive imaging including computed tomography (CT)-based perfusion scan, dynamic contrast-enhanced ultrasonography, dynamic contrast-enhanced magnetic resonance imaging and magnetic resonance imaging-based diffusion-weighted imaging and perfusion (Siegel et al., 2008; Zhu et al., 2008; Lassau et al., 2011; Jiang et al., 2012, 2013; Sahani et al., 2013). CT-based perfusion scans detected significant decreases in tumor perfusion parameters including blood flow, blood volume, and permeability surface area product as well as an increase in mean transit time at 10–12 days after bevacizumab infusion into HCC patients (Zhu et al., 2008; Jiang et al., 2012). Compared to HCC patients with stable disease, those with progressive disease had lower baseline mean transit time and a larger percent increase after bevacizumab treatment (Zhu et al., 2008). Dynamic contrast-enhanced-ultrasonography was shown to quantify tumor vascularity changes as early as 3 days after bevacizumab administration into patients with advanced HCC (Lassau et al., 2011). Importantly, the detected early changes in tumor perfusion had significant correlations with tumor response, PFS, and OS (p = 0.02, 0.02 and 0.003, respectively) (Lassau et al., 2011). Dynamic contrast-enhanced magnetic resonance imaging detected significantly decreased arterial enhancement in HCC lesions after bevacizumab therapy (p = 0.023) (Siegel et al., 2008). Furthermore, in advanced HCC patients treated with sunitinib, an inhibitor to multiple receptor tyrosine kinases, and imaged with diffusion-weighted imaging and magnetic resonance imaging-based perfusion, a larger drop in transfer constant (Ktrans) and redistribution rate constant (Kep) at 2 weeks was correlated with favorable clinical outcome (p < 0.05) (Sahani et al., 2013). In addition, higher baseline Ktrans and larger drop in extracellular volume fraction (EVF) was correlated with longer PFS (p < 0.05) (Sahani et al., 2013). Interestingly, similar imaging biomarkers have also been investigated in patients with recurrent glioblastoma treated with cediranib, an inhibitor of VEGFRs (Batchelor et al., 2007; Sorensen et al., 2009, 2012) or bevacizumab and bevacizumab plus ofranergene obadenovec (Ellingson et al., 2021). These studies demonstrated that increased tumor blood prefusion and vessel normalization were associated with longer PFS and OS. Importantly, the imaging biomarker may also be used to monitor ICI-based immunotherapy as increased vessel perfusion was shown to predict the efficacy of anti-CTLA-4 and anti-PD-1 in tumor-bearing mice (Zheng et al., 2018). Thus, these studies suggest that non-invasive imaging biomarkers should be investigated in atezolizumab plus bevacizumab first-line therapy for predicting treatment responses.

The second type of biomarker is minimally invasive, circulating molecules and cells in a patient's blood. A wide variety of molecules (protein, miRNA, ctDNA) and cells (tumor and immune cells) have been studied as potential circulating biomarkers for anti-angiogenic therapy and ICI-based immunotherapy; however, no definitive circulating biomarkers have been identified for either atezolizumab or bevacizumab in HCC (Boileve et al., 2021). In the single-agent bevacizumab phase II study in patients with advanced HCC, levels of several circulating components including placental growth factor (PGF), VEGF-A, soluble VEGFR-2, and circulating endothelial cells (CECs) were changed (Boige et al., 2012). The following significant correlations were observed between the circulating components and disease outcome: high and increased CEC counts at day 15 were associated with the ORR (p = 0.04) and the 16 week disease control rate (p = 0.02) while lower IL-8 levels at baseline (p = 0.01) and throughout treatment (p ≤ 0.04) were associated with the 16 week disease control rate. Furthermore, high baseline IL-8 and IL-6 levels were associated with shorter PFS and OS(p ≤ 0.04) (Boige et al., 2012). In addition, stromal cell derived factor 1 (SDF-1) and plasma angiogenic activity measured from human umbilical vein endothelial cells decreased upon bevacizumab monotherapy in HCC patients (Siegel et al., 2008). There are limited studies on circulating biomarkers for anti-PD-1/PD-L1 pathway-based therapy in HCC. It appears that high AFP and neutrophil-to-lymphocyte ratio (NLR) in blood are associated with poor outcome while the speed of AFP and protein induced by vitamin K absence-II (PIVKA-II) reductions is associated with OS in anti-PD1 (nivolumab or pembrolizumab) monotherapy of patients with advanced HCC (Kudo et al., 2021; Hung et al., 2021; Teng et al., 2021; Sun et al., 2021). Furthermore, plasma TGF-β (≥200 ng/ml) was associated with poor outcomes after pembrolizumab monotherapy in advanced HCC (Feun et al., 2019). Thus, these readily accessible biomarkers may be used to monitor treatment responses during atezolizumab plus bevacizumab first-line therapy.

The third type of biomarker is derived from analysis of tumor tissues. Although invasive, analysis of HCC tumor tissues has provided important preliminary data to generate hypotheses for large-scale testing of potential biomarkers (Boileve et al., 2021). As demonstrated in the Zhu et al. study, multi-omics (genomic and transcriptomic) analyses of the tumor samples taken prior to therapy revealed that pre-existing immune gene expression signatures, but not tumor mutation burdens, are associated with response to atezolizumab plus bevacizumab first-line therapy in HCC patients (Zhu et al., 2020). The expression levels of PD-L1 in HCC tumors as a predictive biomarker for anti-PD-1 therapy has been evaluated in several clinical trials (El-Khoueiry et al., 2017; Zhu et al., 2018; Sangro et al., 2020a, 2020b). In early CheckMate 040 and KEYNOTE-224 studies, PD-L1 combined positive score (CPS), but not tumor proportional score (TPS), was significantly associated with response rate to anti-PD-1 nivolumab or pembrolizumab monotherapy in patients with advanced HCC (El-Khoueiry et al., 2017; Zhu et al., 2018). However, a follow-up study of CheckMate 040 reported tumor cell PD-L1 expression was significantly associated with improved OS (p = 0.032) (Sangro et al., 2020b). HCC patients with PD-L1 ≥1% on tumor cells had an mOS of 28.1 months vs. 16.6 months for patients with PD-L1 <1% upon nivolumab treatment (Sangro et al., 2020b). In addition, an inflammatory/immune activation gene signature consisting of CD274(PD-L1), CD8A, LAG3, and STAT1 was associated with improved ORR (p = 0.05) and OS (p = 0.01). Furthermore, the phase III CheckMate 459 trial also demonstrated an improved OS for HCC patients with tumor cell PD-L1 > 1% (mOS 16.1 months vs. 8.6 months for sorafenib as first-line) (Sangro et al., 2020a). Thus, although HCC patients respond to anti-PD-1 therapy regardless of their baseline PD-L1 status (El-Khoueiry et al., 2017; Zhu et al., 2018; Sangro et al., 2020a, 2020b), these data support the inclusion of PD-L1 expression levels on tumor and immune cells in HCC tissues as part of a composite biomarker for atezolizumab plus bevacizumab first-line therapy.

A composite biomarker predicting and monitoring responses to the clinical efficacy of both atezolizumab and bevacizumab is likely needed for atezolizumab plus bevacizumab first-line therapy (Fig. 2). The above three types of biomarkers all play important roles in the future composite biomarker: an imaging biomarker examining early changes of tumor vessel perfusion and vascularity in HCC tumors, a circulating biomarker measuring the speed of AFP/PIVKA-II reduction, and tumor-derived biomarkers showing high expression of PD-L1 and immune/inflammation gene signatures at baseline (Fig. 2). A novel class of biomarkers based on gut microbiota of ICI-treated HCC patients is emerging and may become a valuable component of the above composite biomarker (Schwabe and Greten, 2020; Zheng et al., 2019). A key aspect of the composite biomarker is change; continuous monitoring may be needed to detect the dynamics of the components of the composite biomarker. Fortunately, both imaging and circulating biomarkers are readily accessible. Using the longitudinal measurements from the imaging and circulating biomarkers that are continuously monitored, one can build novel predictive models that can provide accurate personalized predictions of risk of target events (e.g., tumor progression, mortality, tumor response) within clinically relevant time windows (e.g., 1 month), in addition to the predictions of these biomarkers' future progression profiles. When new measurements of these biomarkers are available, the predictions can be dynamically updated to reflect patients’ latest prognosis. Based on the accurate predictions, targeted and personalized interventions can be designed to help delay or prevent the onset of unfavorable clinical outcomes (Li and Luo, 2019).

## 6 Summary and future perspective

ICI-based combination immunotherapies have become a major treatment modality for many solid tumors (Table 1). With >2000 clinical trials currently testing ICIs in different combination immunotherapies in cancer treatment (https://clinicaltrials.gov), more treatment combinations will be clinically approved in the near future. We discussed the limitations of the breakthrough atezolizumab plus bevacizumab first-line therapy for HCC as an example of clinically approved combination immunotherapy. Our analysis indicates a need for further patient population selection to maximize benefit and avoid adverse events, a dose optimization for atezolizumab and bevacizumab to provide the correct window of tumor vascular normalization, and establishment of a composite biomarker to guide treatment decisions. These principles likely apply to similar combination immunotherapies in other cancer types.

The future of combination immunotherapies will go far beyond ICI-based therapies as control and eradication of established tumors entail a minimum of four elements: a tumor antigen-targeting antibody, an ICI, a powerful T cell vaccine, and a T cell-stimulating cytokine (Moynihan et al., 2016). Some novel combination immunotherapies have shown promise in preclinical and clinical studies (Yap et al., 2021; Zhu et al., 2021). Inclusion of new classes of immunotherapeutic agents such as neoantigen/tumor-associated antigen-based cancer vaccines is needed in most cancer patients to induce specific anti-tumor immunity (Lu et al., 2021a, 2021b). Furthermore, personalized treatment based on combination immunotherapies is likely the key to control and eradicate established tumors in patients (Wang et al., 2020, 2021).

## CRediT authorship contribution statement

**Ligong Lu:** Conceptualization, were responsible for the conception. **Meixiao Zhan:** Writing – original draft, were drafting and revising the manuscript. **Xian-Yang Li:** Writing – original draft, were drafting and revising the manuscript. **Hui Zhang:** Conceptualization, contributed to conception, discussion and review of the manuscript. **Danielle J. Dauphars:** Writing – review & editing, were reviewing and editing. **Jun Jiang:** contributed to conception, discussion and review of the manuscript. **Hua Yin:** Conceptualization, contributed to conception, discussion and review of the manuscript. **Shi-You Li:** Conceptualization, contributed to conception, discussion and review of the manuscript. **Sheng Luo:** Writing – review & editing, were reviewing and editing. **Yong Li:** Conceptualization, contributed to conception, discussion and review of the manuscript. **You-Wen He:** were responsible for the conception.

## Declaration of competing interest

The authors declare the following financial interests/personal relationships which may be considered as potential competing interests: You-Wen He and Shi-You Li are shareholders of tricision Biotherapeutic Inc.
